# Modification of Immunological Parameters, Oxidative Stress Markers, Mood Symptoms, and Well-Being Status in CFS Patients after Probiotic Intake: Observations from a Pilot Study

**DOI:** 10.1155/2019/1684198

**Published:** 2019-11-23

**Authors:** Letizia Venturini, Sara Bacchi, Enrica Capelli, Lorenzo Lorusso, Giovanni Ricevuti, Chiara Cusa

**Affiliations:** ^1^Department of Internal Medicine and Therapeutics, Cellular Pathophysiology and Clinical immunology Laboratory, University of Pavia, 27100, Italy; ^2^Department of Earth and Environmental Sciences, University of Pavia, 27100, Italy; ^3^U.O: Neurology, A.S.S.T. San Leopoldo Mandic Hospital of Merate, 23807, Italy

## Abstract

The present study discusses about the effects of a combination of probiotics able to stimulate the immune system of patients affected by Chronic Fatigue Syndrome/Myalgic Encephalomyelitis (CFS/ME). To this purpose, patients diagnosed according to Fukuda's criteria and treated with probiotics were analyzed by means of clinical and laboratory evaluations, before and after probiotic administrations. Probiotics were selected considering the possible pathogenic mechanisms of ME/CFS syndrome, which has been associated with an impaired immune response, dysregulation of Th1/Th2 ratio, and high oxidative stress with exhaustion of antioxidant reserve due to severe mitochondrial dysfunction. Immune and oxidative dysfunction could be related with the gastrointestinal (GI) chronic low-grade inflammation in the lamina propria and intestinal mucosal surface associated with dysbiosis, leaky gut, bacterial translocation, and immune and oxidative dysfunction. Literature data demonstrate that bacterial species are able to modulate the functions of the immune and oxidative systems and that the administration of some probiotics can improve mucosal barrier function, modulating the release of proinflammatory cytokines, in CFS/ME patients. This study represents a preliminary investigation to verifying the safety and efficacy of a certain combination of probiotics in CFS/ME patients. The results suggest that probiotics can modify the well-being status as well as inflammatory and oxidative indexes in CFS/ME patients. No adverse effects were observed except for one patient, which displayed a flare-up of symptoms, although all inflammatory parameters (i.e., cytokines, fecal calprotectin, ESR, and immunoglobulins) were reduced after probiotic intake. The reactivation of fatigue symptoms in this patient, whose clinical history reported the onset of CFS/ME following mononucleosis, could be related to an abnormal stimulation of the immune system as suggested by a recent study describing an exaggerated immune activation associated with chronic fatigue.

## 1. Introduction

Chronic Fatigue Syndrome/Myalgic Encephalomyelitis (CFS/ME) is a pathologic condition characterized by persistent and unexplained relapsing fatigue, physical and cognitive, that is worsened by physical and mental exertion [1]. It is a multisystemic condition in which immune functions, mitochondrial function, and cardiovascular, neurological, and endocrine systems are compromised [2–4]. The estimated prevalence of CFS/ME in the worldwide population is between 1 and 0.4% with an age onset of 30-40 years and a prevalence in female subjects [5, 6], and increasingly frequently, also children are diagnosed with CFS/ME, probably as a result of an increased dissemination of knowledge about the disease [7]. Since specific markers for this disease are yet unknown, consequently, the diagnosis is carried out according to the exclusion criteria. It is possible that in the past, many CFS/ME cases were unrecognized or that incorrect diagnoses have been made. For example, a diagnosis of mental and behavioral disorder for patients with CFS/ME is not uncommon, supported by the observation that these patients often complain difficulty of concentration or look like they have depression [8].

CFS/ME is a condition that is very debilitating both in terms of severity and duration of illness [9, 10]. Since the first description, many researchers have carried out studies in various directions to search for the possible etiologic factors, for example, the role of infectious or chemical and/or physical agents. The infectious hypothesis is based on the observation that CFS/ME often arises following virus infection or reactivation (i.e., Epstein Barr Virus (EBV)). In favor of the toxic hypothesis, there are a lot of data in the literature describing CFS/ME onset after exposure to toxic agents [11, 12]. All researchers agree with the occurrence of an impaired immune response in CFS/ME, particularly as concerns Natural Killer (NK) cell activity [13, 14]. Moreover, a modification in the T-helper cell 1 (Th1)/T-helper cell 2 (Th2) balance leading to an increase in Th2 activation profile, a decreased cytotoxic activity of neutrophils, an altered functionality of B cell subpopulations, and a different ability in metabolizing xenobiotic agents has been described [15–18]. But the main hypothesis about the aetiology of CFS/ME is the infectious one, based on observation that the onset is frequently associated with EBV infection or reactivation.

Patients with CFS/ME exhibit an abnormal immune response to exercise, oxidative stress system dysfunction, and modification in the gene expression profile of immune cells [19]. The increased expression of toll-like receptor 4 correlated with postexertional malaise, which is the main feature of ME/CFS, suggests the existence of an impaired interaction between these immune receptors and pathogenic or xenobiotic agents [20].

Moreover, in CFS patients, structural and functional mitochondrial dysfunctions are documented which lead to increased oxidative stress indexes in response to exercise or inflammation and exhaustion of antioxidant reserve; amelioration of symptoms might occur in relation to antioxidant administration [21–28]. This immune and oxidative dysfunction is associated with gastrointestinal (GI) chronic low-grade inflammation in the lamina propria and intestinal mucosal surface, leaky gut, bacterial translocation, and a particular microbiota composition, overall promoting further immune and oxidative dysfunction. As reported in the literature, bacterial species can modulate the functions of the immune and oxidative systems and the administration of some probiotics can improve mucosal barrier function, modulating the release of proinflammatory cytokines, in CFS/ME patients [21–31]. Chronic inflammation within the gut exaggerates enteric autonomic activation and impairs the anti-inflammatory cholinergic system leading to systemic low-grade neuroendocrine-immune activation that in turn induces a vicious circle, an additional intestinal inflammation and dysfunction as well as the trigger of all inflammatory systemic cascade responsible of CFS/ME symptomatology. It has been also suggested that CFS/ME could be an autoimmune disease for the frequently described relapsing-remitting course and for a higher prevalence in females than in males, which suggests a different gene regulation under the influence of sex hormones [32–34]. The hypothesis of an immune deregulation and of a disrupted tolerance might be related with gastrointestinal (GI) disturbances, frequently observed in CFS/ME patients [35]. The key for the interpretation of this disease may reside in the tolerance mechanisms, in which mesenteric lymph nodes (MLNs) exert a central role in addition with the signals from commensal microbiota [36]. Most lymphocytes and antibodies are produced in the gut, and MLNs are localized at a pivotal area for the control of immune anatomy and migration, forming the crossing border between mucosal immunity and the remainder immune system. The imbalance between Th1 and Th2 immune response observed in CFS/ME could be ascribed to changes in the intestinal barrier functions, which in turn, could trigger autoimmune processes [37]. In support of this hypothesis, patients with this syndrome often complain gastrointestinal disorders with the persistence of a low-grade inflammation in the lamina propria of the intestinal mucosa [38]. It is widely reported that CFS/ME arises from, and is perpetuated by, a mucosal deregulated immune response triggered by unknown etiological factors in a genetically susceptible individual. Emerging research studies on the microbial flora of CFS/ME patients have reported a different fecal microbial composition with a reduction of *E. coli* and Bifidobacterium spp. and a significant rise in the *Enterococcus* sp. prevalence [39]. Similarly, in the small and large intestines of patients with chronic enteritis, a decrease of Bifidobacterium spp. was observed [40]. The gut microbiota plays a major role in the immune system functioning: in general, wealth and diversity of bacterial species in the ecosystem bowel are considered indicators of good health [41, 42]. By contrast, a reduced bacterial biodiversity characterizes different pathological situations, such as inflammatory bowel disease, allergic diseases, type 2 diabetes, and autism [42–45]. Some studies claim that microbial diversity within the gut is also positively associated with mental well-being [46]. Several clinical studies suggest that probiotic administration could represent a preventive and therapeutic strategy for allergic and chronic inflammatory diseases for their capacity to modify the gut microbial composition, improve mucosal barrier function, and downregulate proinflammatory cytokines [47]. In recent years, the knowledge about gut microbiota has been largely improved and the ability of some cultivated bacterial species to modulate the functions of the immune system has been demonstrated [48, 49]. Among these, lactobacilli act as immunoregulators through interaction of lipoproteins with toll-like receptor 2 (TLR2) and of peptidoglycans with nucleotide-binding oligomerization domain-containing protein 2 (NOD2) [50]. In the hypothesis that an altered gut microbiota with a mucosal barrier dysfunction and an aberrant intestinal immunity are involved in the pathogenesis of CFS/ME, a modification of gut microbiota could be one strategy to control the development and/or progression of this disorder. Therapeutic target of probiotic administration in CFS is multisystemic. Probiotics improve mucosal barrier functions reducing low-grade chronic inflammation as well as bacterial translocation. They modulate the gut-brain neuroendocrine axis which benefits the autonomic nervous system (ANS) inflammatory vicious circle leading to improvement in mood symptoms, pain sensitivity, and cognitive functions. Moreover, they seem to reduce oxidative stress indexes, improve antioxidant defense and mitochondrial function, and modulate immune system response [50]. With the aim to evaluate the effectiveness of some cultivated probiotics to modulate immune functions and counteract the symptoms of CFS/ME, we conducted a randomized clinical trial on patients diagnosed according to Fukuda's criteria treated with probiotics known to have a therapeutic effect in experimental animal models of autoimmune encephalomyelitis [51].

## 2. Materials and Methods

### 2.1. Study Population

Patients with CFS/ME diagnosis according to Fukuda's criteria referred to the clinic of Internal Medicine and Geriatrics ASP-IDR Santa Margherita (Pavia, Italy) who gave their consent to participate in the trial were enrolled for the study. The exclusion criteria were as follows: lack of consent, overlap with other diseases, early diagnosis of psychiatric illness, somatoform disorders, abuse of alcohol and drugs, antibiotic therapy, and presence of risk factors for probiotic sepsis according to Boyle's criteria [52].

The study was approved by the local Ethics Committee, and before starting treatment, all the patients signed a written consent to participate in the study in accordance with the Declaration of Helsinki (1964).

### 2.2. Probiotics

Four different mixtures of probiotics (Bromatech s.r.l, Milano, Italy) were employed for treatments: *Enterococcus faecium* and *Saccharomyces boulardii* (Enterelle); *Bifidobacterium longum*, *Bifidobacterium breve*, *Bifidobacterium bifidum*, and *Bifidobacterium infantis* (Bifiselle); *Bifidobacterium longum AR81* (Rotanelle); *Lactobacillus casei* and *Bifidobacterium lactis* (Citogenex); and *Lactobacillus rhamnosus* GG and *Lactobacillus acidophilus* (Ramnoselle) according to the schedule of Table 1.

All patients were treated for eight weeks. Probiotics were administered to patients without modifying their usual diet. Nonpathogenic *E. coli* and some fungi (i.e., *Saccharomyces boulardii*) have been demonstrated to be beneficial in CFS/ME patients [29].

Probiotic mixture was selected in relation to the literature data in order to positively interfere in clinical history of CFS/ME.

The probiotics were selected due to their previous documented pharmacological actions in order to affect the intestinal dysbiosis, to reinforce the intestinal mucosal barrier, and to modulate the immune system. The probiotics selected specifically improve mucosal barrier function (mucus and tight junction formation) [53, 54] and compete with gut preexistent pathogens to enhance gut permeability and low-grade gut inflammation and to reduce bacterial translocation. Modulation of the gut immune (GALT) and systemic immune systems depends on probiotic interaction with TLRs activating dendritic cells to trigger modulation of polarizing program (which is different in relation to probiotic combination) to stimulate T1 or regulatory T cell profile [55, 56].

The consequent reduced local and systemic inflammation improves the immune and oxidative system functions [57, 58] and affects the modulation of gut-brain interactions with mood leading to better cognitive performances [51, 59, 60]. The literature suggests that administration of *L. casei*, *L. acidophilus*, and *B. lactis* leads to cognitive improvement in CFS/ME patients [51]. High dose of *L. casei* strain Shirota (LcS) significantly increases both fecal lactobacillus and Bifidobacterium spp. and is associated with anxiety reduction in CFS/ME, suggesting their effect on gut-brain axis modulation with psychological benefit [61].

### 2.3. Laboratory Analysis

The *erythrocyte sedimentation rate (ESR)* was assessed by a modified Westergren Method: venous blood samples (5–10 mL) were taken in vacutainer tubes under sterile conditions from patients and controls between 08:30 and 10:30 am. Serum was obtained from freshly drawn and rapidly centrifugated. Serum was quickly frozen at −70°C and stored until processed.

*Reactive oxygen metabolites* were determined photometrically by performing the d-ROM test (Diacron International, s.a.s., Grosseto, Italy) on heparinized plasma.

For *immunophenotyping of leukocytes*, fresh blood samples were collected by venipuncture in EDTA separator tubes and promptly applied to peripheral blood mononuclear cell (PBMC) isolation by Ficoll Density Gradient, using LSM 1077 Lymphocyte Separation Medium (PAA, Pasching, Austria) and centrifugation at 2200 rpm for 20 minutes at 20°C. The intermediate layer consisting of PBMC was recovered, washed in Hanks's medium (PAA) containing 0.1% BSA and 0.5 mM EDTA, and stained with monoclonal antibody against CD3, CD4, CD8, and CD19 (MBL International, Woburn, MA). Flow cytometric analysis was performed through an Epics XL cytometer (Beckman Coulter).

For *quantitative serum immunoglobulin test*, serum fraction of peripheral blood samples was obtained to detect the levels of the three major classes of immunoglobulin (IgG, IgA, and IgM). Ig concentrations were measured by nephelometric technique using the BN Prospec Nephelometer Analyzer and commercially available kits from Dade Behring, Marburg, Germany.

*Urinary free cortisol (UC)* was measured in the urine collected over 24 hours, making a night dexamethasone suppression test, by liquid chromatography-tandem mass spectrometry (LC-MS/MS) method.

*Determination of dehydroepiandrosterone sulfate (DHEA-S) concentration* was measured by chemiluminescence technique on the automatic device “Immulite 2000” Siemens® Los Angeles, CA, USA.

For *determination of fecal calprotectin (CAL)*, the stool samples were prepared and analyzed for calprotectin levels according to the manufacturer's instructions (PhiCal Calprotectin ELISA Kit; Immunodiagnostic, Bensheim, Germany). Calprotectin levels, expressed as micrograms per gram of feces, were determined in a stool homogenate obtained with the addition of an extraction buffer containing citrate in a weight/volume ratio of 1 : 50 with the quantitative ELISA method at OD 450 nm.

For *determination of C-reactive protein (CRP)*, quantitative concentration of CRP was determined in serum fraction by immunoturbidometric method by means of the Abbott Architect c-800 system and using 6K26 MULTIGENT CRP Vario (Abbott Laboratories, Illinois, USA).

### 2.4. Statistical Analysis

Statistical analysis was performed with the commercial software SPSS (for Windows, version 20.0; SPSS Inc., IBM, Armonk, NY, USA) according to the appropriate tests for each considered variable. One-way analysis of variance was applied for comparison among groups. Tukey's test and Bonferroni's corrections were used as post hoc tests. The *t*-test was applied for means comparisons. *p* values less than 0.05 will be considered significant.

### 2.5. Clinical Evaluations

Health status, quality of life, and mood were assessed both at the beginning and at the end of the study administering a short battery of 4 questionnaires. The *SF-36 Health Survey* is a polyvalent short form health survey composed of 36 questions that yields two summary indexes, physical and mental, respectively, PCS and MCS [62]. *Chadler's scale* is used to measure the severity of fatigue [63]. The *Beck Depression Inventory I (BDI-I)* and *Beck Depression Inventory II (BDI-II)* are used for measuring the severity of depression [64, 65].

## 3. Results

The present study was carried out from January 2010 to January 2014 at the Internal Medicine and Geriatrics Department of Pavia University at the ASP-IDR Hospital Santa Margherita (Pavia, Italy). 13 patients diagnosed with ME/CFS who met the criteria for CFS/ME defined by Fukuda's criteria were enrolled [1]. Four of 13 recruited patients discontinued treatment early, before the 8-week protocol of going out of the study. The statistical analysis was performed on 9 of 13 patients enrolled. All eligible patients gave consent to diet supplementation with probiotics, according to the protocol described in Materials and Methods.

The probiotics used to treat patients were chosen to consider the possible pathogenic mechanisms of CFS/ME syndrome which has been associated with an impaired immune response against a hypothetical infectious agent and with a dysregulation of Th1/Th2 ratio. Each patient received a probiotic combination of different types of bacteria able to counteract pathogens, reinforce the mucosal barrier, and modulate the immune system. More specifically, the probiotic mixture (Enterelle) is composed of *Enterococcus faecium UBEF-41*, *Saccharomyces cerevisiae* sub*. boulardii*, and *Lactobacillus acidophilus LA 14*. *E. faecium* and *S. boulardii* are bacterial strains with a competitive action against antibiotic-resistant microorganisms such as *E. coli*, *C. difficile*, and *C. albicans*. Particularly, *S. boulardii* stimulates IgA production [66]; *L. acidophilus* regulates dendritic cell activation and maturation [67]. The other probiotic mixtures contain different Bifidobacterium spp.: *B. longum*, *B. breve*, *B. bifidum*, *B. infantis* (Bifiselle), and *B. longum AR81* strain (Rotanelle).

Bifidobacteria are anaerobic microorganisms that colonize the intestine and counteract proliferation and metabolic activities of other bacteria helping with the removal of nitrogen compounds derived from putrefaction processes triggered by the proteolytic bacteria *Klebsiella*, *Proteus*, *Clostridia*, and *Bacteroides* [68]. Moreover, a lot of the metabolites produced by Bifidobacteria are short-chain fatty acid able to stimulate the immune system and induce the differentiation of dendritic cells. In particular, *B. longum* stimulates Th1 subset and antiviral action and *B. bifidum* stimulates T-helper cell 17 (Th17) subset [69–71]. *Lactobacillus casei* was demonstrated to be able to rebalance Th1/Th2 subsets and stimulate the innate immune response mediated by NK cells and macrophages through the stimulation of IL-10 and IL-12 secretion [72].

In order to obtain a multifunctional stimulation and a functional rebalancing of the immune system, two different types of probiotic preparations were administered to CFS/ME patients: *Lactobacillus rhamnosus* combined with *Lactobacillus casei* (Ramnoselle) and *Lactobacillus casei* combined with *Bifidobacterium lactis* (Cytogenex). Clinical evaluations to assess well-being status and laboratory tests to state inflammatory indexes were performed before and after probiotic administrations.

**Figure 1:**
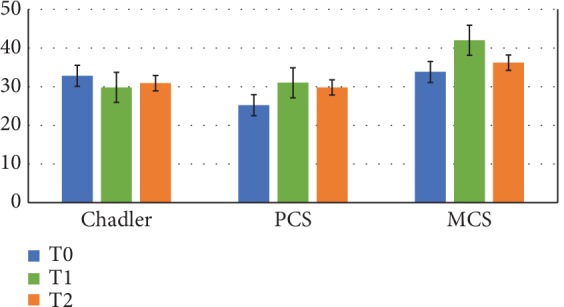
Health status indexes. Chadler's scale score and PCS and MCS indexes, respectively, for physical and mental condition before and after probiotic protocol. T0: mean basal values; T1: mean values after 4 weeks of probiotic protocol; T2: mean values after 8 weeks of probiotic protocol (*n* = 9).

The evaluation of well-being status was performed by applying Chadler's scale and the Short Form Health Survey (SF-36), before and after probiotic intake to assess, respectively, physical (PCS) and mental (MCS) components. In Figure 1, the mean values of PCS and MCS obtained by applying the SF-36 survey are shown, before, during, and after taking the probiotic protocol. Comparing the indexes obtained during and after probiotic intake with the basal values, we observed a progressive reduction of Chadler's scale score indicating a reduction of fatigue and a progressive increase of both PCS and MCS indexes indicating an improvement of both physical and mental conditions after probiotic administration. All these results agree with an overall increase in the quality of life of patients.

**Figure 2:**
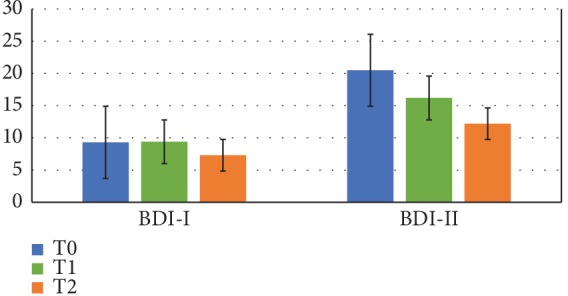
Mood indexes. Mean values of Beck Depression Inventory tests (BDI-I and BDI-II) before and after probiotic protocol. T0: mean basal values; T1: mean values after 4 weeks of probiotic protocol; T2: mean values after 8 weeks of probiotic protocol (*n* = 9).

In Figure 2, the values obtained by applying the two different Beck Depression Inventory (BDI-I and BDI-II) tests are reported. The results of both tests showing a reduction of indexes during and after probiotic protocol in comparison with the basal values indicate an improvement of mood according to the reduced perception of fatigue observed by means of PCS tests.

**Figure 3:**
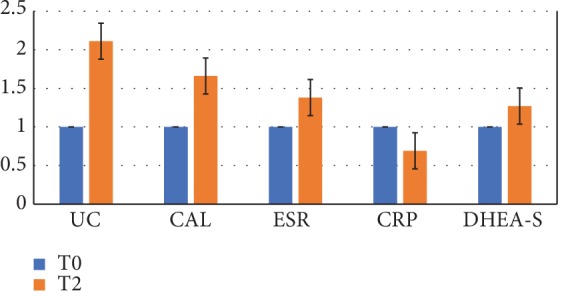
Inflammatory parameters. Inflammatory indexes (fold change values) following probiotic administration. UC: urinary free cortisol; CAL: fecal calprotectin; ESR: erythrocyte sedimentation rate; CRP: C-reactive protein; DHEA-S: dehydroepiandrosterone sulfate (*n* = 9).

In order to evaluate the probiotic capacity to modify inflammatory chronic condition, a series of analyses evaluating different indexes were carried out; specifically, urinary cortisol (UC), fecal calprotectin (CAL), erythrocyte sedimentation rate (ESR), and C-reactive protein (CRP) were measured. The relative increases/decreases of the fold change values after taking probiotics with respect to the baseline were calculated for each parameter, and the mean values obtained were compared. The results are reported in Figure 3, showing the increase of urinary free cortisol (2.3x), ESR (1.7x), and DHEA-S (1.4x) and a reduction of about 30% of CRP values after probiotic intake. The results obtained indicated that although CRP reduces after probiotic intake, other inflammatory indexes increase at the end of the study versus baseline. The differences were not statistically significant.

It is well known that the ESR index is vulnerable to misinterpretation in clinical practice unlike CRP, which is sensitive to subtle changes in the acute phase response and falls quickly once inflammation subsides, because of its short half-life. For this reason, in chronic inflammatory conditions, the accuracy and sensitivity of ESR and CRP is a topic of debate because of age, gender, and adiposity. For example, high ESR/low CRP discordance is frequently observed in women, likely associated with their propensity to develop connective tissue disorders as reported by some studies [73].

Our data shows that the more sensitive test for systemic inflammation (CRP) ameliorates after probiotic administration.

As concerns calprotectin, which is a specific marker of intestinal inflammation, patients showed basal values higher than the normal range (2.5-10x), with an increase of these values after probiotic treatment. The fecal calprotectin (CAL) increase does not agree with systemic inflammatory index (CRP) reduction as well as with the improvement in clinical symptoms (such as mood improvement and reduction in fatigue symptoms) observed; it is possible that probiotic administration modulating the inflammatory, immune, and nervous enteric systems might initially induce a local and transient increase of local inflammation; further data and/or successive controls are necessary to better clarify the significance of the data obtained.

Urinary free cortisol (UC) as well as DHEA-S increases after probiotic intake; it must be noted that the levels of urinary cortisol as well of DHEA-S are very low in the CFS/ME patients studied (in accordance with the literature data) and that the level of urinary cortisol is within the normal range after probiotic administration. Cortisol and DHEA-S levels are closely linked with stress as well as with the body's ability to cope with stressful conditions; moreover, it has to be noted that patients with CFS/ME usually exhibit low cortisol levels. The increase (to normal) of these hormones we observed seems to indicate the ability of probiotic administration to interfere with the neuroendocrinology of stress, increasing stress hormone production; moreover, amelioration of stress response might be associated with the obtained clinical data in terms of reduction of fatigue and amelioration of mood symptoms as well as quality of life.

**Figure 4:**
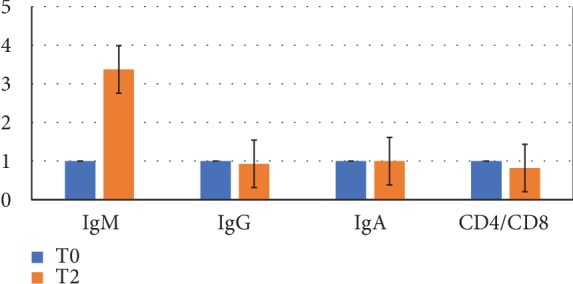
Immunological parameters. Immunoglobulin levels and CD4+/CD8+ lymphocytes ratio (fold change values) following probiotic intake (*n* = 9).

To evaluate the ability of probiotics to stimulate the immune system, serum levels of immunoglobulins (IgM, IgG, and IgA) and the prevalence of CD4 and CD8 lymphoid cell subsets were determined. In Figure 4, the average values of these parameters (fold change values) after probiotic intake are shown. Considering the mean values, after treatment with probiotics, a significant increase of IgM (of about 3x over the basal values) was observed, but no changes as concerns IgG and IgA serum levels were observed; further data and/or successive controls are necessary to better clarify the significance of the data obtained. A reduction of CD4/CD8 ratio with a mean index value of 1.78 vs. 2.06 was obtained. Three patients showed a higher reduction (more than 50%), 1 patient a slight reduction (about 5%), and in 5 patients, this index was unchanged.

**Figure 5:**
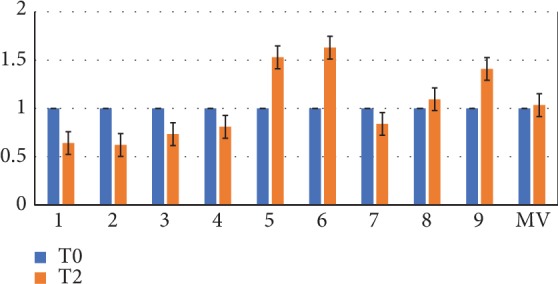
Oxidative stress. Oxidative stress index (d-ROMs) in each patient following probiotic intake. T2 levels compared to T0 levels (baseline) (fold change values, T0/T2 ratio). MV: mean values (*n* = 9).

In Figure 5, the d-ROM index before and after probiotic protocol is reported. Similarly to CD4/CD8 ratio, a slight reduction of mean values was obtained but a great variability among patients.

**Figure 6:**
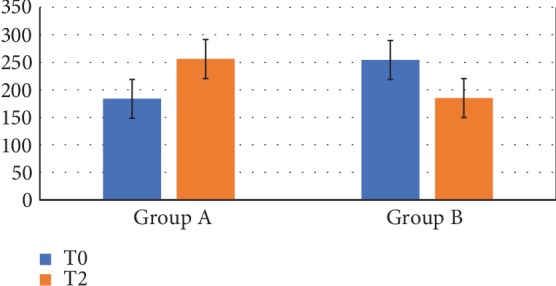
Oxidative stress index (d-ROMs) in the two groups of patients at T0 and at T2. Group A: very low d-ROM values at T0; Group B: normal d-ROM values at T0 (*n* = 9). Patients with very low d-ROM values in T0 (Group A) increase oxidative production in T2; patients with normal d-ROM values at T0 (Group B) decrease oxidative production after probiotic intake.

Particularly, patients with very low d-ROM values in T0 (Group A) increase oxidative production in T2; conversely, patients with normal d-ROM values at T0 (Group B) decrease oxidative production after probiotic intake as reported in Figure 6.

**Figure 7:**
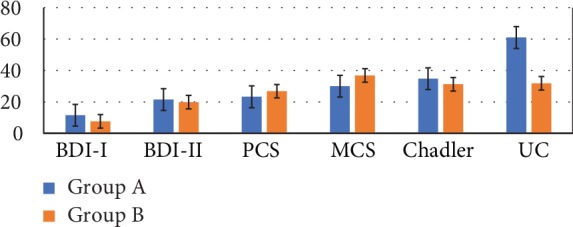
Inflammatory parameters, health status indexes, and mood indexes. Group A at T0 and Group B at T0 compared (*n* = 9). Lower d-ROM in T0 is associated with greater degree of depression (BDI) and fatigue (Chadler's scale score), higher levels of UC, and lower physical and psychological quality of life (PCS and MCS lower level). UC level is higher in Group A than in Group B in T0.

Comparison between Group A and Group B at T0 (Figure 7) indicates that patients with lower d-ROM values in T0 have greater degree of depression (higher levels in BDI tests) and greater symptoms of fatigue (higher Chadler's scale score). Moreover, Group A shows higher levels of UC and lower physical and mental indexes of quality of life (higher levels in SF-36 indexes both in PCS and MCS) than patients in Group B.

**Figure 8:**
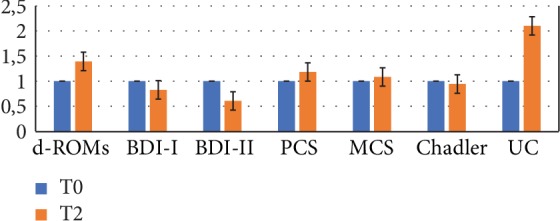
Inflammatory parameters, health status indexes, and mood indexes. Group A at T0 and at T2 compared (fold change values, T0/T2 ratio) (*n* = 9).

Following probiotic intake, amelioration of fatigue (Chadler's score), improvement of mood and quality of life indexes (MCS and PCS), reaching significant values for mental health, and urinary cortisol level increase were observed in Group A as suggested by fold change values by comparing T0 and T2 results (Figure 8, fold change values, T0/T2 ratio).

**Figure 9:**
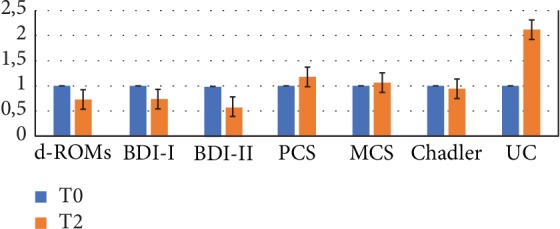
Inflammatory parameters, health status indexes, and mood indexes. Group B at T0 and at T2 compared (fold change values, T0/T2 ratio) (*n* = 9).

Except for d-ROM levels, after taking the probiotic protocol, similar results were obtained in Group B, as shown in Figure 9 (fold change values, T0/T2 ratio) where we reported the fold change values obtained by comparing T0 and T2 results of Group B.

To explain the individual difference in the effects of probiotic intake, we analyzed the possible correlation between d-ROM changes and psychophysiological states of CFS/ME patients. Our results suggest a nonsignificant correlation between d-ROM and Chadler's scale (*p* = 0.346, *t* = 1.0097), between d-ROM and BDI-I and BDI-II inventory (*p* = 0.389, *t* = 0.91882; *p* = 0.184, *t* = 1.4734, respectively), and between d-ROM and PCS indexes (*p* = 0.708, *t* = 0.39086). Interestingly, the analysis between d-ROM and MCS indexes shows a significant correlation with *p* = 0.043 and *t* = 2.4659 underlying the potential probiotic beneficial effect on mood and psychological state. The data obtained show that patients with very low levels of stress oxidative response (lower d-ROM basal levels) increase oxidative stress after therapy but exhibit a similar trend of clinical response to probiotic administration. Further data and/or successive controls are necessary to better clarify the significance of the data obtained.

## 4. Discussion

The changes in clinical and laboratory features show a modulation of intestinal and systemic inflammation as well as an improvement in fatigue and mood symptoms after the probiotic protocol supplementation in patients enrolled. The results suggest that the probiotic ability to counteract the main features of CFS/ME, i.e., chronic fatigue, immune imbalance, and psychophysical discomfort, affects the well-being status of patients. Our data agreed with a recent study which demonstrated that some bacterial strains belonging to the Bifidobacterium genus improve mood and quality of life in patients with inflammatory bowel disease (IBD); the improvements were associated with changes in brain activation patterns indicating a reduction of limbic reactivity due to probiotic activity [74].

Probiotics modulate the host's defenses including the innate and acquired immune system [75] playing an important role in the prevention and in the therapeutic approach of infectious diseases and of chronic inflammation, particularly of the digestive tract. A lot of studies, particularly those based on the most advanced technologies, have demonstrated that probiotics can interfere with commensal and/or pathogenic microorganisms because of synergic and antagonist mechanisms between different bacterial groups [76]. This interaction is crucial during prevention and treatment of infections to gut microbial equilibrium retrieval. Probiotics also act on microbial products like toxins on host products (e.g., bile salts) and food components resulting in toxin inactivation and host detoxification. The use of probiotics aims at stabilizing or reconstituting the physiological balance between the intestinal microbiota and its host. However, it must be stressed that no specific probiotic is able to prevent or treat all kinds of diseases, because the ability of probiotics depends on the kind of molecules expressed in the gut of patients and on the metabolic properties of components secreted by the bacterial strains. The main target cells are gut epithelial and gut-associated immune cells. The interaction of probiotics with host cells by adhesion itself might already trigger a signaling cascade leading to immune modulation. Alternatively, release of soluble factors can trigger signaling cascades in immune or epithelial cells.

The manipulation of the gut flora cannot however be considered a precision intervention because of the lack of knowledge about microbial communities that harbor the human gut and, particularly, on the complex relationships among the different species. Finally, although probiotics are considered safe, there are concerns about their use in people with highly compromised immune systems, and in premature infants, as revealed by some infection events as a consequence of probiotic intake in immunosuppressed children and in severely debilitated patients [52, 77, 78]. Although these events occurred rarely, it is necessary to take into account the possibility of bacterial translocation in the presence of an increased intestinal permeability frequently occurring in gut inflammation and of an incomplete, not yet well-established, microbial colonization, which represents a common condition for newborns and preterm infants [79]. On the other hand, the evidence for the efficacy of probiotic supplementation demonstrated well-established benefits [80].

The observations here reported and obtained from a pilot study aimed at exploring the feasibility of an adjuvant treatment in CFS/ME patients. Even with a low sample size, our results showed that in CFS/ME patients, the administration of some combination of probiotics could be practicable and safe. The probiotics used were able to improve psychophysical well-being of patients. No adverse effects were observed in all patients. One patient showed an exacerbation of symptoms at the beginning of the therapy, and for this reason, the treatment was discontinued. It was not possible to ascertain whether this event was related to the assumption of probiotics. We were not able to ascertain whether this effect was a consequence of the treatment or due to an intrinsic factor of that subject such as the preexistence of an overactive immune system. A recent published study highlighted that a chronic fatigue condition with diagnostic features overlapping those observed in CFS/ME condition arises in a small proportion of patients who were treated with interferon alpha to stimulate the immune system [81]. Considering that CFS/ME is a condition in which the immune system seems to be constantly subjected to activation stimuli, it is worthwhile, for further studies, to consider the possibility that an exaggerated stimulation of the immune system could cause a worsening of symptoms. To avoid this event, a larger monitoring of the immune activation status of patients to be treated and the choice of those probiotics having a prevalent immune regulation activity, avoiding those with high stimulatory effects, would be desirable.

In the context of a low sample size, the data reported here show that treatment with probiotics can be viable and safe. The clinical laboratory data obtained are representative of a population of patients with CFS/ME treated with a combination of probiotic microorganisms administered at an appropriate dosage and chosen according to their specific therapeutic targets.

## Figures and Tables

**Table 1:** Probiotic protocol.

1st week	Enterelle 2 cps bid
2nd week	Bifiselle 2 cps bid
3rd week	Ramnoselle 2 cps bid
4-8th week	Enterelle 2 cps Citogenex 2cp Rotanelle 2 cps

## Data Availability

The excel data used to support the findings of this study are available from the corresponding author upon request.
